# STAT3 is involved in miR-124-mediated suppressive effects on esophageal cancer cells

**DOI:** 10.1186/s12885-015-1303-0

**Published:** 2015-04-19

**Authors:** Yan Cheng, Yang Li, Yuanyuan Nian, Dong Liu, Fei Dai, Jun Zhang

**Affiliations:** Department of Digestive Diseases, The Second Affiliated Hospital, Medical School of Xi’an Jiaotong University, Xi’an, Shaanxi 710004 PR China; Department of Otolaryngology-head and neck surgery, The Second Affiliated Hospital, Medical School of Xi’an Jiaotong University, Xi’an, Shaanxi 710004 PR China

**Keywords:** miR-124, Esophageal cancer, STAT3, Malignant phenotype

## Abstract

**Background:**

Esophageal cancer (EC) is one of the most common cancers worldwide. The cancer-related inflammation pathway- signal transducer and activator of transition 3 (STAT3) signaling pathway has been reported to play critical role in its initiation and progression, while the way mediated its hyperactivation remains elusive so far. Accumulating studies reported the important role of microRNAs (miRNAs) in the regulation of gene expression, among of which, the miR-124/STAT3 interaction has been widely reported in various cancers, while its role in EC has not been investigated yet.

**Methods:**

Firstly, we identified the target role of STAT3 in esophageal cancers using Dual-luciferase reporter assays. Next, we explored the expression of miR-124 in EC tissues. To further investigate its effects on the malignant phenotype of EC cells, we completed a series of experiments. Through transfection with miR-124 mimic, the expression of miR-124 in esophageal cancer cell lines, Eca109 and TE-1, were restored. Next, we detected the effects of ectopic miR-124 expression on the proliferation, cell cycle distribution, apoptosis, migration and invasion of EC cells *in vitro*, and the tumor growth *in vivo*.

**Results:**

Dual-luciferase assays identified that STAT3 is a target gene of miR-124 in esophageal cancer cells. Over-expression of miR-124 significantly down-regulated the mRNA and protein levels of STAT3. Moreover, we found that the expression of miR-124 was consistently suppressed in esophageal cancer tissues and cell lines. Next, functional experiments showed that ectopic expression of miR-124 in EC cells induced a complex phenotype, namely an inhibition of cell proliferation, block of G1/S phase transition, induction of cell apoptosis, and suppression of cell invasion *in vitro*, as well as inhibition of tumor growth *in vivo*. Moreover, restored the expression of STAT3 in esophageal cancer cells transfected with miR-124 before, could partially abolished the suppressive effects of miR-124 on the proliferation and invasion of Eca109 cells.

**Conclusion:**

Collectively, these data suggest that miR-124 functions as a tumor suppressor in esophageal cancer through, at least partially, targeting STAT3 signaling pathway.

## Background

Esophageal cancer is one of the most common cancers worldwide, which is ranked eighth in incidence and sixth in mortality [1]. Since the 1990s, its morbidity and mortality among the world rose steadily, especially in the rural areas. The etiology of this neoplasm is complex. In addition to the genetic and environmental factors, diet and lifestyle also contribute to the complicate scenario, which results in a deficiency of internationally accepted standard prevention and chemotherapy regimen [2]. Thus, a comprehensive understanding of the biology about this malignancy is definitely necessary to the development of novel therapies.

Currently, the role of chronic inflammation in the esophageal carcinogenesis has been widely explored [3]. A key concept of the cancer-related inflammation pathway is that some genetic events endow cancer cells with growth advantages [4], among of which, an important one is the signal transducer and activator of transcription-3 (STAT3) signaling pathway [5]. STAT3 is a critical member of the STAT transcription factor family. Activation by tyrosine phosphorylation leads to its dimer formation, translocation to the nucleus, recognition of STAT3-specific DNA-binding elements, and transcriptional activation of the target genes [6]. Thus, by targeting various genes STAT3 has been reported to participate in a variety of physiological progresses, such as cell proliferation, apoptosis and so on [7,8]. For esophageal cancer, STAT3 was constitutively activated in cancer tissues [9], and overexpression of STAT3 could activate esophageal epithelium cells to form tumors *in vivo* by up-regulating Oct-1 [10]. Otherwise, its suppression was also investigated to be involved in metformin-mediated autophagy and apoptosis promotion of esophageal squamous cell carcinoma [11]. In view of the critical role of STAT3 in esophageal carcinogenesis, the way mediated its hyperactivation remains elusive so far.

Nowadays, accumulating studies reported the important role of microRNAs (miRNAs) in the regulation of gene expression. MiRNAs represent a group of endogenous, small, non-coding RNAs [12], which induce various target genes translational silence or cleavage by partially complementing with the 3’-untranslated region (3’UTR) of specific messenger RNAs [13]. The interplay between miRNAs and STAT3 signaling pathway has been widely studied [14]. Among of these miRNAs, miR-124, which is a kind of highly conserved miRNA, attracted our attention. In addition to regulating nervous system development [15], it also acts as a tumor suppressor, as well as an independent prognostic marker for many kinds of cancers [16,17]. STAT3 has been reported to be the target gene of miR-124 in endometrial cancer cells, and be involved in the miR-124-mediated suppressive effects on endometrial cancer cells [18]. Strikingly, rs531564 GG polymorphism of primary gene of miR-124, pri-miR-124-1 which may promote the expression of miR-124, has been observed to show significant effects on decreasing the risks of esophageal squamous cell carcinoma in subgroups of elderly persons, females, no drinking and no smoking Chinese people [19]. These make us speculate that miR-124 might function as a potential tumor suppressor in esophageal caner, and STAT3 signaling pathway might be involved in the suppressive effects.

Thus, in this study, we firstly explored the expression of miR-124 in 67 paired esophageal cancer tissues, and then investigated its effects on the malignant phenotype of esophageal cancer cells. Then, we further explored whether the effects of miR-124 on cell proliferation and invasion are mediated by *STAT3*.

## Methods

### Tissue specimens

67 formalin fixed paraffin-embedded specimens of esophageal cancer tissues were collected from department of Digestive Diseases, the Second Affiliated Hospital of Medical School of Xi’an Jiaotong University. The matched normal tissues were obtained from the 5 cm distant from the tumor margin, which were further confirmed by pathologists. All patients did not perform any therapy before recruitment to this research. The use of the tissue samples for all experiments were obtained with informed consent and approved by the Second Affiliated Hospital of Medical School of Xi’an Jiaotong University institutional Ethics Committee.

### Cell culture and transfection

Human normal esophageal cell line Het-1A and three human esophageal cancer cell lines (Eca109, Ec9706 and TE-1) were obtained from the American Type Culture Collection (ATCC, Manassas, VA, USA). These cells were maintained in Dualbecco’s modified Eagle’s medium (DMEM, Invitrogen, CA, USA) supplemented with 10% fetal bovine serum (FBS; PAA, Pasching, Austria) and streptomycin (100 μg/mL), penicillin (100 U/mL). Cultures were incubated in a humidified atmosphere of 5% CO_2_ at 37°C. Transfection of oligonucleotides were performed as previously supplemented [20]. MiR-124 and relative scramble mimic were purchased from Dharmacon (Austin, TX, USA). According to manufacturer’s instructions, all oligonucleotides were transfected into ECa109 and TE-1 cells to a final concentration of 50 nM by Dhamafect 1 (Dharmacon, Lafayette, CO, USA). Cells were collected for further experiments 48 h post-transfection.

### RNA extraction, reverse transcription and quantitative real-time PCR

According to the protocol of Recover All Total Nucleic Acid Isolation Kit (Ambion, Austin, TX, USA), total RNA was isolated from 20-μm sections from formalin-fixed, paraffin-embedded tissue blocks. The expression level of U6 and *GAPDH* was regard as an internal control of miRNAs and mRNA, respectively. Total RNA was reversely transcribed using First-Strand cDNA Synthesis kit (Invitrogen, Carlsbad, CA, USA) with specific primers qualified with a Taqman probe. Then, quantitative real-time PCR was performed to quantify relative expression of miRNA and mRNA using the Quanti-TectSYBR Green PCR mixture on an ABI PRISM 7900 Sequence Detection System (Applied Biosystems, Carlsbad, CA, USA). The primers used for reverse transcriptions and quantitative RT-PCR were summarized in Table 1. The relative expression levels were evaluated using the 2^-△△Ct^ method.

**Table 1:** **Oligonucleotide primer sequences for PCR or Reverse transcription**

**Gene**	**Primer sequence**
Primers for reverse transcription	
miR-124	5′-GTCGTATCCAGTGCAGGGTCCG AGGTATTCGCACTGGATACGA
	CGGCATTC-3′
U6	5′-AAAATATGGAACGCTTCACGAATTTG-3′
*STAT3*	5′-TTTTTTTTTTTTTTTTTT-3′(Oligo(dT))
*GAPDH*	5′-TTTTTTTTTTTTTTTTTT-3′(Oligo(dT))
Primers for quantitative real-time PCR	
miR-124-F	5′- GGACTTTCTTCATTCACACCG-3′
miR-124-R	5′- GACCACTGAGGTTAGAGCCA-3′
U6-F	5′-CTCGCTTCGGCAGCACATATACT-3′
U6-R	5′-ACGCTTCACGAATTTGCGTGTC-3′
*STAT3-*F	5′-GAAGGACATCAGCGGTAAGA-3′
*STAT3*-R	5′-AGATAGACCAGTGGAGACAC-3′
*GAPDH*-F	5′-TCAACGACCACTTTGTCAAGCTCA-3′
*GAPDH*-R	5′-GCTGGTGGTCCAGGGGTCTTACT-3′
Primers for *STAT3* PCR amplifying	
*Up-stream*	5′-TGACTCCCTTTCTCCCTGG-3′
*Down-stream*	5′-GAACTGAATGAAGACGCCAT-3′

F for forward, R for reverse.

### Plasmid construction and luciferase reporter assays

The Eca109 and TE-1 cells were seeded in triplicate in 24-well plates and allowed to settle for 12 h. The whole 3’-UTR of *STAT3* gene was cloned and amplified. Mutation in 3’-UTR of *STAT3* gene with miR-124 putative target binding site deleted was generated with the QuickChange Site-Directed Mutagenesis kit (Stratagene, CA, USA). Both the wild and mutant *STAT3* genes were cloned into the pGL-3-vector (Promega, Wisconsin, USA) immediately downstream of the *Renilla* luciferase gene. A luciferase reporter construct containing the miR-124 consensus target sequence served as the positive control (PC) and the pRL-TK vector was used as positive and internal controls (PC), respectively. Cells were co-transfected with pGL-3 firefly luciferase reporter (50 ng), pRL-TK Renilla luciferase reporter (10 ng) and miR-124 (50nM) or scramble mimic (50nM) with Lipofectamine 2000 (Invitrogen, Carlsbad, CA, USA). Cell lysates were prepared using Passive Lysis Buffer (Promega, Wisconsin, USA) 48 h upon transfection, and luciferase activity was measured using the Dual-Luciferase Reporter Assay (Promega, Wisconsin, USA). Results were normalized to the Renilla luciferase.

### CCK-8 assays

The Cell Counting Kit-8 (CCK-8, Dojindo, Kumamoto, Japan) Assays were performed to explore the effects of miR-124 on the proliferation of Eca109 and TE-1 cells. 5 × 10^3^ cells were plated into 24-well plates upon transfection with miR-124 mimic. The CCK-8 reagents were added to the each wells at 0 h, 24 h, 48 h, and 72 h post-transfection, and cells were diluted in normal culture medium at 37°C until visual color conversion occurred. The absorbance values in each well were measured with a microplate reader set at 450 nm and 630 nm.

### FACS analysis

For analysis of cell apoptosis, Eca109 and TE-1 cells were collected and diluted to a concentration of 1 × 10^6^ cells/ml and washed with ice-cold PBS three times 72 h after transfection. Cells were incubated with PE Annexin-v and 7AAD according to the PE Annexin v Apoptosis Detection Kit I(BD Pharmingen, CA, USA) protocol. For analysis of cell cycle distribution, cells were harvested 48 h upon transfection with miR-124 mimic. Cells were washed twice with cold PBS, fixed in ice-cold 70% ethanol, and incubated with propidium iodide (PI) and RNase A. Cells harvested in two experiments were all analyzed by fluorescence-activated cell sorting (FACS). Data were analyzed with Flowjo software.

### Cell migration and invasion assays

Migration assays were carried out in modified Boyden chambers (BD Biosciences, San Jose, CA, USA) with 8 μm pore filter inserts in 24-well plates. 24 hours after transfection, 2 × 10^5^ cells suspended in serum-free DMEM were added to the upper chamber. While for invasion assays, the transwell chambers were coated with Matrigel (BD Biosciences, San Jose, CA, USA) before, and 4 × 10^5^ cells were added to the upper chamber after 24 h of transfection. DMEM containing 20% FBS were added to the lower chambers as a chemoattractant. After 24 h incubation, the non-filtered cells in both assays were gently removed with cotton swabs. Filtered cells located on the lower side of the chamber were stained with crystal violet, air dried and photographed.

### Immunoblot analysis

For the Immunoblot assays, cells were harvested in ice-cold PBS 48 h after transfection and lysed on ice in cold modified radioimmunoprecipitation buffer supplemented with protease inhibitors. Upon protein concentration was determined using the BCA Protein Assay Kit, equal amounts of protein were analyzed by SDS-PAGE. Gels were electroblotted onto nitrocellulose membranes (Millipore, Wisconsin, USA). After blocked with 5% non-fat dry milk in Tris-buffered saline containing 0.1% Tween-20 2 h, membranes were incubated at 4°C over night with primary antibodies (STAT3, p-STAT3, Bcl-xL, MMP-9 and GAPDH, Cell Signaling, Massachusetts, USA). Then, membranes were incubated with respective second antibodies and detected by peroxidase-conjugated secondary antibodies using the enhanced chemiluminescence system (ECL) (Millipore, Wisconsin, USA).

### In vivo studies

Animal xenograft model studies were performed according to institutional guidelines; 4 × 10^6^ Eca-109 cells were inoculated subcutaneously in posterior flanks of 6-week-old female nude mice, four mice per group. When tumors reached 100 mm^3^, miR-124 mimic and relative scramble mimic diluted in lipofectamine 2000 solution (100 nmol mimic in 100 μl total volume) were injected directly into the tumors, respectively. The tumors were injected every 4 days for a total of six times. Tumor diameters were measured after 10 days from injection and then every three days. After 28 days after injection, mice were killed and tumors were weighted after necropsy. Tumor volume was calculated as follows: length × width^2^ × 1/2. All animals received humane care in compliance with the Public Health Service Policy on Humane Care and Use of Laboratory Animals. The use of animals for all experiments were obtained with informed consent and approved by the Second Affiliated Hospital of Medical School of Xi’an Jiaotong University institutional Ethics Committee.

### Statistical analysis

Data were expressed as the mean ± standard deviation of at least three independent experiments. Statistical analysis was carried out using the Student’s t-test for comparisons of two groups, unless otherwise indicated (χ^2^ test), and data with three groups were analyzed using a one-way analysis of variance (ANOVA). Statistical analysis was carried out using SPSS 15.0 software. *P*-values < 0.05 were considered significant.

## Results

### MiR-124 directly targets STAT3 in esophageal cancer cells

**Figure 1:**
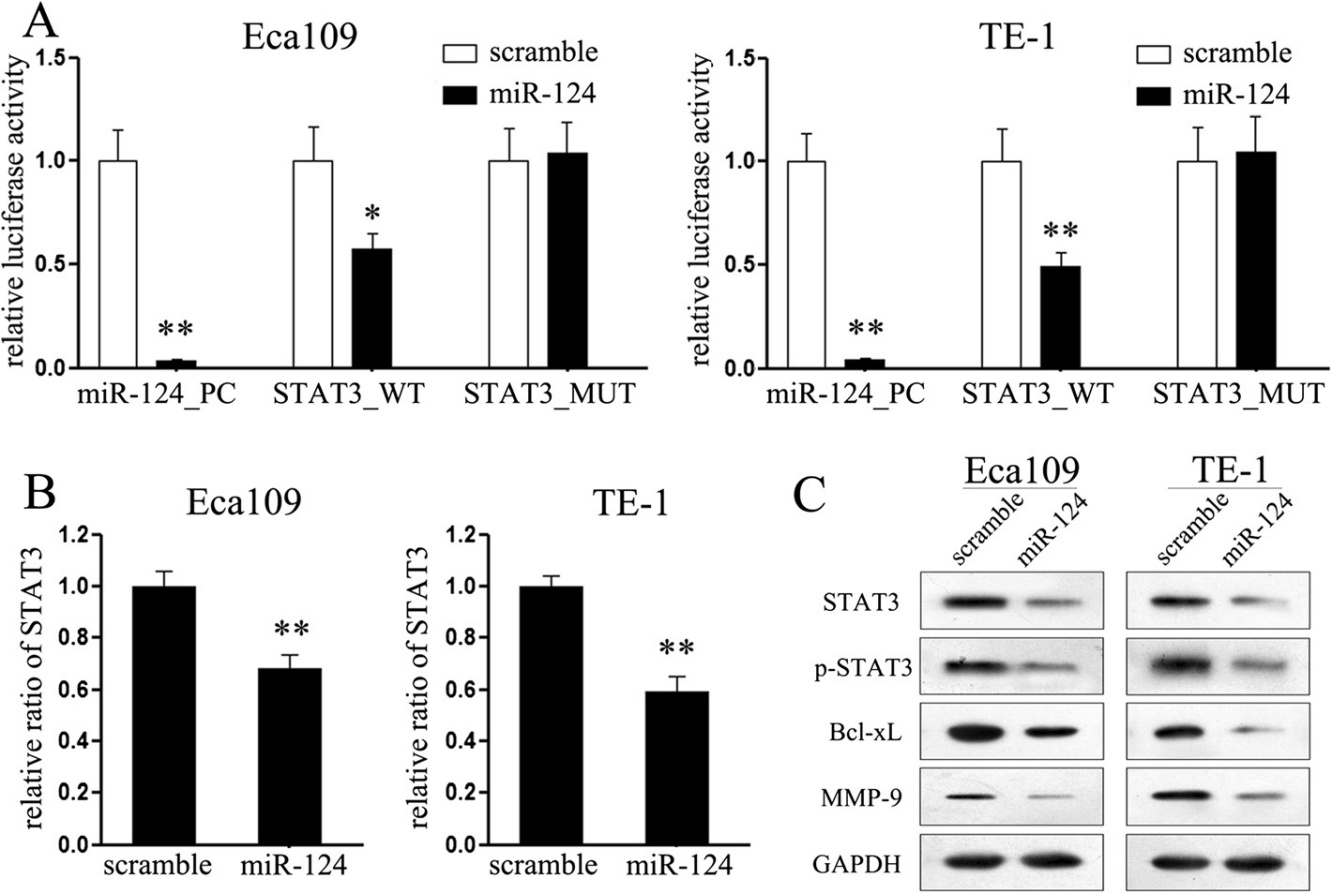
MiR-124 targets *STAT3* gene in esophageal cancer cells. **(A)** Relative luciferase activity of the indicated STAT3 reporter construct in both esophageal cancer cell lines, co-transfected with miR-124 mimic or scramble mimic, is shown; **(B)** Quantitative RT-PCR assays were performed to detect the expression of *STAT3* upon transfection with miR-124 mimic or scramble mimic (normalized to *GAPDH*); **(C)** Western blot analysis showed the expression levels of STAT3, p-STAT3, Bcl-xL and MMP-9 proteins in esophageal cancer cells upon transfection with miR-124 mimic. **P < 0.01.

Although a previous work has reported that STAT3 signaling pathway is involved in miR-124-mediated tumor suppression on endometrial cancer cells, it remains open whether or not STAT3 is also be its putative gene in esophageal cancers. According to the putative binding site of miR-124 in the 3’UTR of *STAT3* gene previously reported [18], luciferase reporter assays were performed. Co-transfection with miR-124 and constructs containing the 3’UTR of miR-124 putative binding site led to significant suppression of luciferase activity in both esophageal cancer cell lines (Figure 1A), suggesting that miR-124 suppressed the transcription activity of *STAT3* gene in esophageal cancer cells by targeting the putative 3’UTR of *STAT3* mRNA independently. Identical to the luciferase reporter assays, we observed the consistently decreased expression of mRNA and protein of *STAT3* upon transfection with miR-124 mimic in both cell lines (Figure 1B and C). Otherwise, since *STAT3* has been reported to participate in variety of biological progresses by targeting different down-stream genes, such as *Bcl-xL* and *MMP-9* [21-23], we also explored the effects on the expression of these genes upon transfection. As expected, ectopic expression of miR-124 suppressed the expression of phosphorylated STAT3 at tyrosine 705 (termed p-STAT3) and its downstream genes, Bcl-xL and MMP-9 protein at same time. Collectively, these findings identified that miR-124 regulates the expression of *STAT3* post-transcriptionally in esophageal cancer cells.

### MiR-124 expression was consistently suppressed in esophageal cancer tissues and cell lines

**Figure 2:**
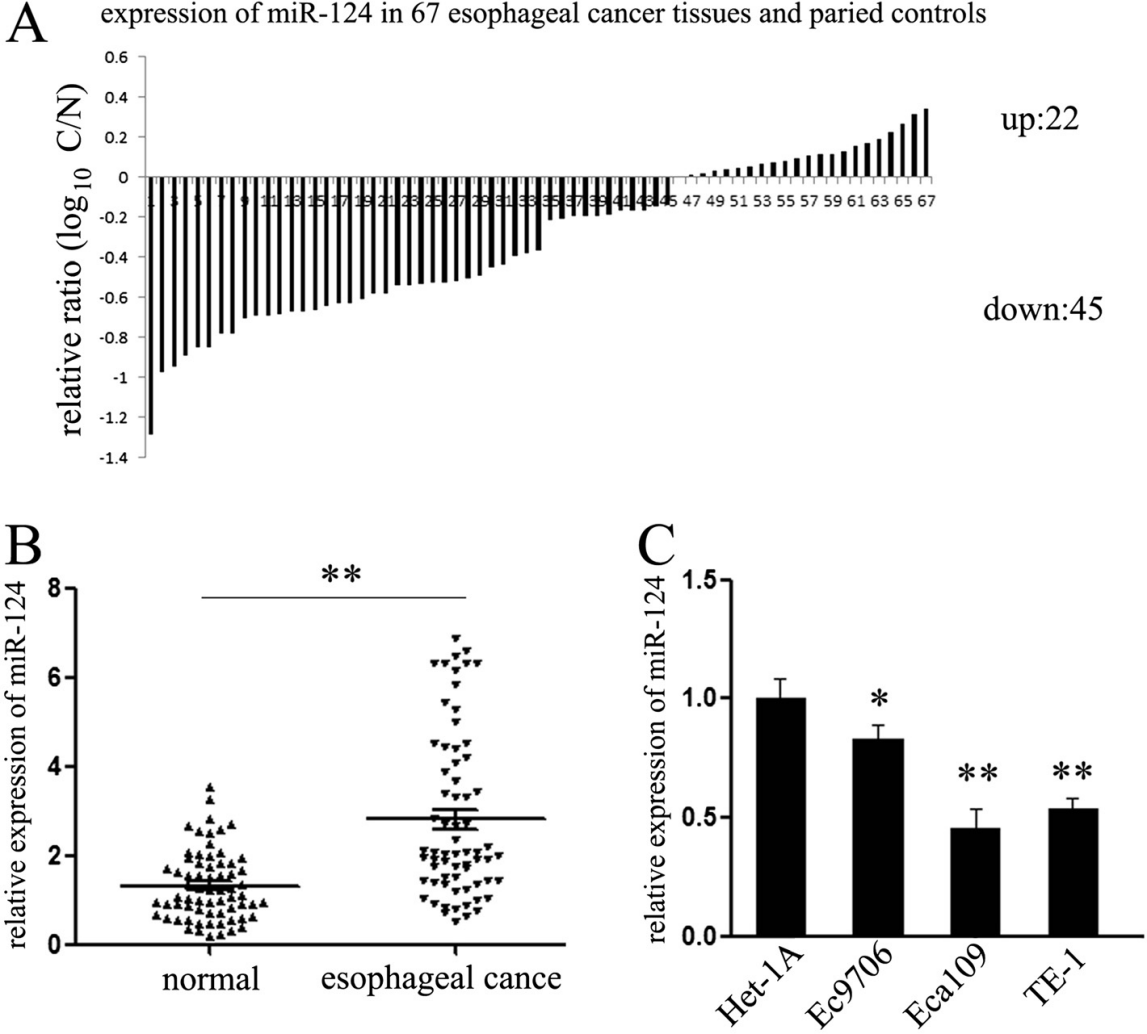
Expression level of miR-124 in esophageal cancer tissues and cell lines. **(A)** The expression of miR-124 in 67 pairs of esophageal cancer tissues and compared normal tissues was detected using TaqMan quantitative RT-PCR. Data are shown as log_10_ of relative ratio change of esophageal cancer tissues relative to normal tissues; **(B)** Statistical analysis of relative miR-124 expression levels in esophageal cancer tissues and compared normal tissues; **(C)** Using quantitative RT-PCR analysis, the expression of miR-124 in four esophageal cancer cell lines (Eca109, Ec9706 and TE-1) was analyzed relative to normal esophageal cell line Het-1A. The expression of miR-124 were normalized to small nuclear RNA U6. **P < 0.01.

The fact that *STAT3* signaling pathway has been widely reported to be activated in esophageal cancer tissues, and perform important function in the initiation and progression of this tumor make us to speculate that whether miR-124 was involved in its regulation and function. In an attempt to explore the expression and significance of miR-124 in esophageal carcinogenesis, we firstly detected the expression of miR-124 in 67 pairs of esophageal cancer tissues and adjacent normal tissues (Table 2). As shown in Figure 2A, relative to the adjacent normal tissues majority, about 67% (45 out of 67), of selected esophageal cancer tissues exhibited under-expression of miR-124. In order to observe the tendency of miR-124 expression intuitively, we further performed the statistical analysis the miR-124 expression, which indicated that the expression of miR-124 is much lower in cancer tissues compared with the normal tissues (Figure 2B). The generality of this observation was further confirmed in esophageal cancer cell lines. Comparing with the human normal esophageal cell line Het-1A, the expression of miR-124 is consistently down-regulated in three different esophageal cancer cell lines (Eca109, Ec9706 and TE-1) (Figure 2C). These data suggested that alteration of miR-124 might be a frequent event in human esophageal cancer and has a pivotal role in the tumorigenesis of esophageal cancer.

**Table 2:** **Relationship between miR-124 expression and their clinicopathological parameters in 67 esophageal cancer patients**

**Clinicolpathological parameters**	**Number of cases**	**Expression of miR-124**	***P*** **-value**
Age (years)			
<60	38	1.301 ± 0.8616	0.8220
≥60	29	1.344 ± 0.6687	
Gender:			
Male	52	1.267 ± 0.7489	0.3017
Female	15	1.504 ± 0.8765	
Tumor size (cm):			
≤4	45	1.344 ± 0.8002	0.7154
>4	22	1.270 ± 0.7485	
Degree of differentiation:			
Well and moderately	27	1.590 ± 0.8159	0.0182*
Poorly	40	1.137 ± 0.7052	
Local invasion:			
T1 + T2	24	1.604 ± 0.6710	0.0245*
T3 + T4	43	1.161 ± 0.7965	
TNM stage:			
StageI + II	42	1.434 ± 0.8117	0.1192
Stage III + IV	25	1.127 ± 0.6930	
Metastasis:			
No	41	1.362 ± 0.7076	0.5831
Yes	26	1.253 ± 0.8900	

*P*-value represents the probability from a Student’s t-test for miR-124 expression between variable subgroups. *P < 0.05 was considered to have a significant difference.

### MiR-124 overexpression suppresses proliferation and induces apoptosis of esophageal cancer cells

**Figure 3:**
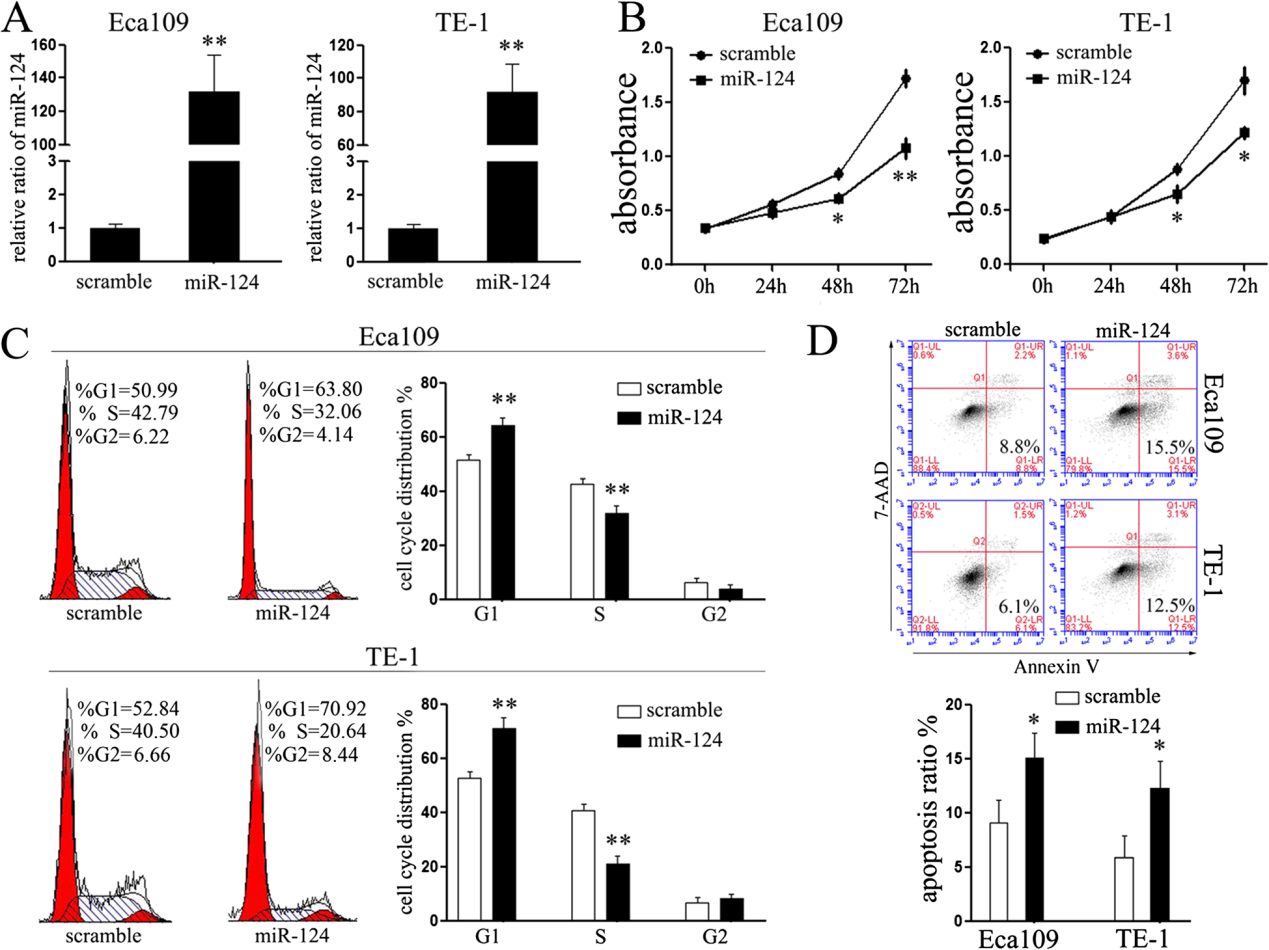
MiR-124 suppresses esophageal cancer cell growth. **(A)** RT-PCR was performed to detect the expression of miR-124 in esophageal cancer cell lines (Eca109 and TE-1) after treatment with miR-124 mimic (normalized to U6); **(B)** CCK-8 assays were performed to analyze the effect of miR-124 on cell proliferation of Eca109 and TE-1 cells; **(C, D)** The influences of miR-124 on cell apoptosis and cell cycle progression were analyzed using fluorescence-activated cell sorting (FACS); *P < 0.05; **P < 0.01.

As miR-124 expression consistently decreases in esophageal cancer tissues and cells, we sought to compensate for its loss through exogenous transfection with miR-124 mimic into Eca109 and TE-1 cells. Upon transfection, the intracellular levels of miR-124 were about 130-fold and 90-foud higher in Eca109 and TE-1, respectively (Figure 3A). Then, we explored the effects of miR-124 on the proliferation and apoptosis of these two cell lines. As expected, ectopic expression of miR-124 led to significant decrease in cell proliferation in both esophageal cell lines (Figure 3B). Cells transfected with miR-124 showed a significant decrease in the percentage of cells in S phase (*P* < 0.01) and an increase in the percentage of cells in G1 phase (*P* < 0.01) (Figure 3C). Furthermore, we explored the biological role of miR-124 on the apoptosis of Eca109 and TE-1 cells using the PE Annexin V staining assays. Cells undergoing early apoptosis bind only to annexin V, and cells binding both are either in the late stages of apoptosis or already dead. As shown in Figure 3D, ectopic miR-124 expression increased proportions of annexin V –positive only cells compared to scramble control group (*P* < 0.05) (Figure 3D), suggesting miR-124 can efficiently induce apoptosis of esophageal cancer cells.

### Ectopic expression of miR-124 significantly impairs the migratory capacity of esophageal cancer cells

**Figure 4:**
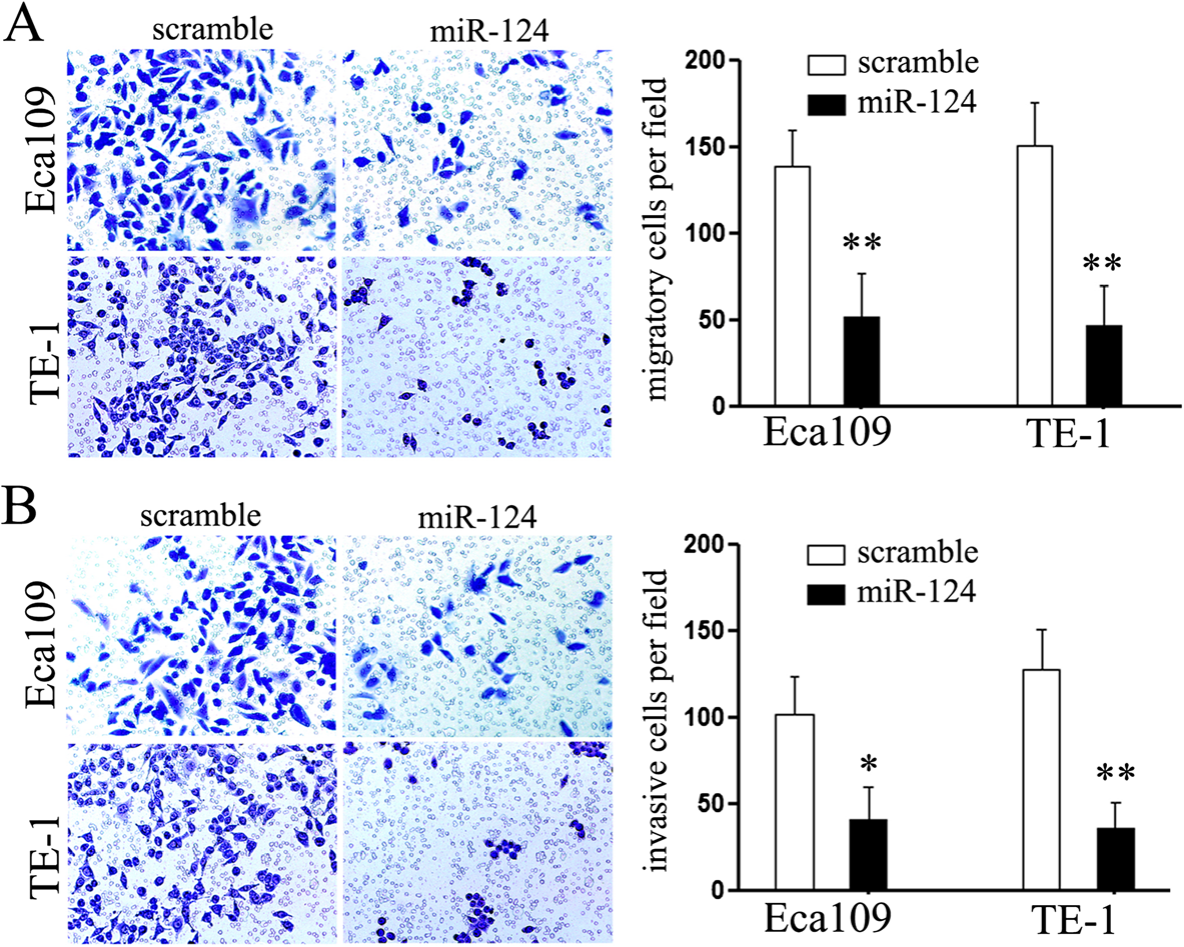
MiR-124 inhibits cell migration and invasion. **(A, B)** The effects of miR-124 on cell migration and invasion were detected using transwell chamber assays. Panel **A** showed the results on migration; Panel **B** showed the results on invasion. The chambers have been coated with Matrigel, which functions as the extracellular cell matrix. MiR-124 inhibited cells invasion through the membrane. *P < 0.05; **P < 0.01.

Considering the important role of metastasis in the tumor progression, we sought to further evaluate the effects of miR-124 on the migratory and invasive capacity of Eca109 and TE-1 cells using Matrigel migration and invasion assays, respectively. For both experiments, cells were maintained in serum-free medium during the course of assays to avoid any augmented migratory behavior that could be affected by cell proliferation. Firstly, we found that re-expression of miR-124 in TE-1 and Eca109 cells resulted in a significant reduction in cell migration compared with the control groups (*P* < 0.01) (Figure 4A). For invasive assays, the transwell chambers were coated with Matrigel that mimics the extracellular matrix. As shown in Figure 4B, transfection with miR-124 significantly suppressed cells passing through the chambers coated with Matrigel, which means miR-124 significantly suppressed the invasive capacity of TE-1 and Eca109 cells. Taken together, these results indicated a significant role of miR-124 on repressing cell motility and invasiveness of esophageal cancer cells *in vitro*.

### STAT3 is involved in miR-124-mediated tumor suppression

**Figure 5:**
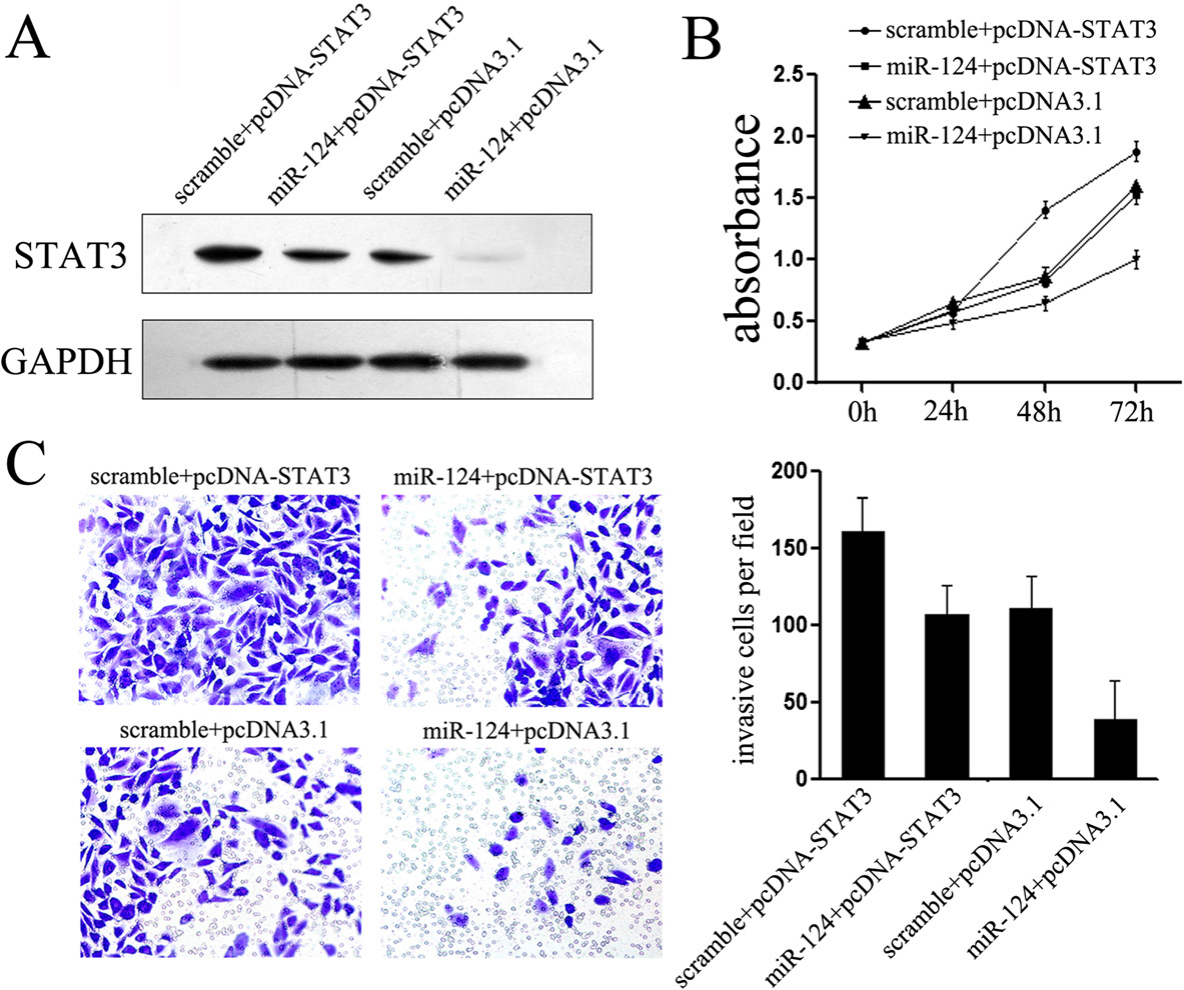
MiR-124 suppresses tumor progression through targeting *STAT3* in esophageal cancer cells. **(A)** Upon transfection with *STAT3* construct, we rescued the expression of *STAT3* in Eca109 cells; **(B)** CCK-8 assays were used to detect to explore the effects of miR-124/*STAT3* interaction on cell proliferation; **(C)** Transwell assays were performed to detect the effects on cell invasion of Eca109 cells treated as described in B. *P < 0.05; **P < 0.01. Data are presented as means ± SD. Statistical analysis was carried out using ANOVA.

The results mentioned above strongly suggested the tumor suppressor role of miR-124 in esophageal cancer, while the role of STAT3 in miR-124-mediated suppressive effects remains unknown. To further explore whether miR-124-mediated growth inhibition in esophageal cancer cells via the direct targeting of *STAT3*, we adopted a “rescue” methodology. We generated a new construct containing the full ORF of *STAT3* gene (pcDNA3.1-*STAT3*). As expected, the expression of *STAT3* was rescued when pcDNA3.1-*STAT3* was transfected into Eca109 cells that had been treated with miR-124 mimic before (Figure 5A). In agreement with the restored expression of *STAT3* protein, increased cell proliferation (Figure 5B) was observed upon transfection with pcDNA3.1-*STAT3.* Moreover, restored expression of *STAT3* also partially abolished the suppressive effects of miR-124 on cell invasive capacity (Figure 5C). These data established the participation of *STAT3* in miR-124 pathway, i.e. the tumor suppressor role of miR-124 in esophageal cancer might be typically a consequence of decreased *STAT3* expression.

### MiR-124 inhibited the growth of Eca-109-engrafted tumors

**Figure 6:**
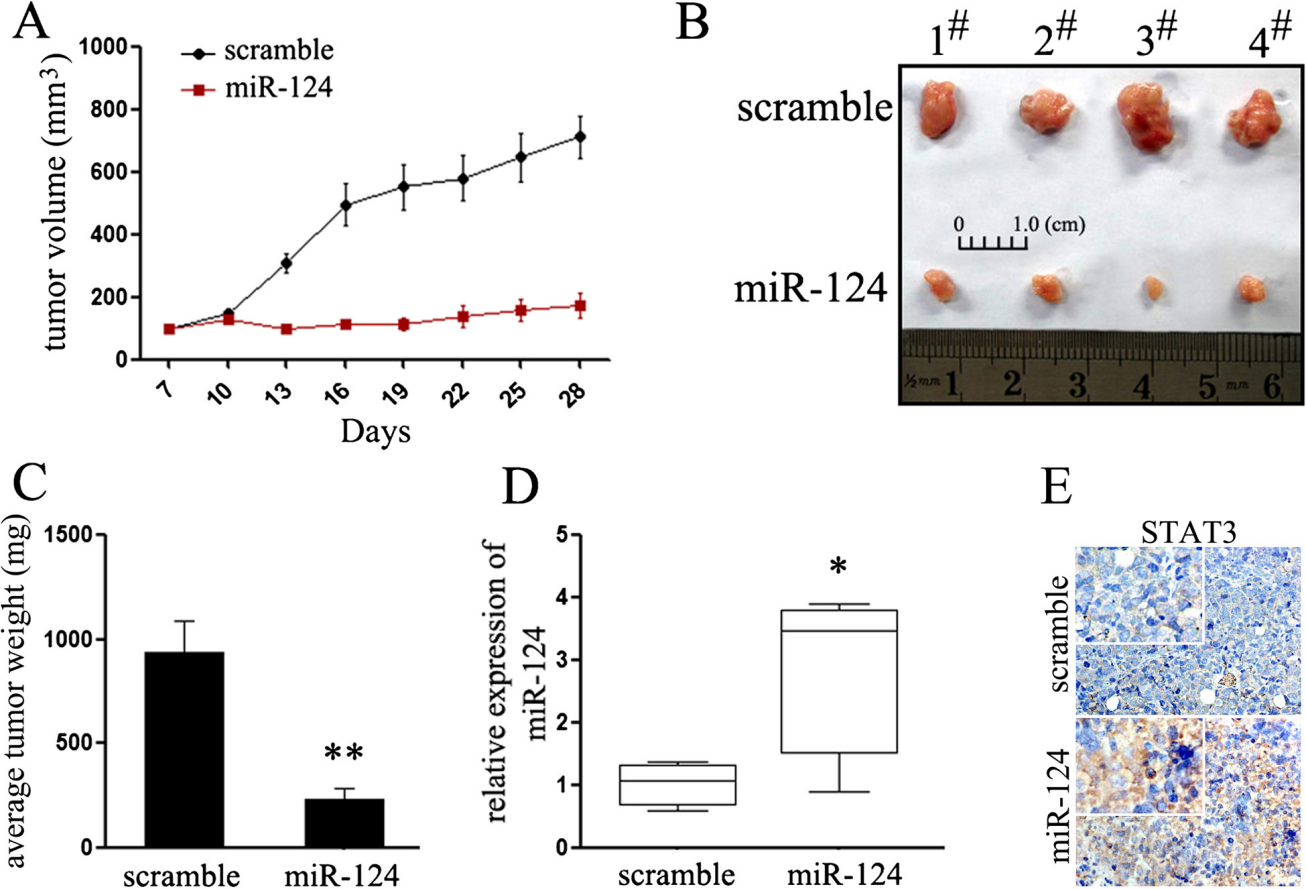
MiR-124 inhibits esophageal cancer growth in vivo. **(A)** Graphic representing tumor volumes at the end of the experiment for mice treated with miR-124 mimic or scramble mimic. Four mice per group; **(B)** Tumor volume averages between scramble and miR-124 mimic treated mice groups at the indicated days during the experiment; **(C)** Tumor weight averages between scramble and miR-124 mimic treated mice groups at the end of the experiment (28 days); **(D)** Quantitative RT-PCR analysis showed the relative expression of miR-124 in injected tumor tissues (normalized to U6); **(E)** Immunochemistry analysis showed the expression of STAT3 in injected tumor tissues. **P < 0.01.

Our above findings indicated that miR-124 was potential therapeutic targets in esophageal cancer. To further explore the therapeutic effect of miR-124 on esophageal tumorigenicity *in vivo*. 4 × 10^6^ Eca-109 cells were inoculated subcutaneously in posterior flanks of immunocompromised “nude” mice. When tumors reached 100 mm^3^, synthetic miR-124 or scramble mimic were injected into the tumors. After six consecutive injections, we found that injection with miR-124 inhibited the growth of Eca-109-engrafted tumors with respect to scramble mimic-treated tumors (Figure 6A, 6B). Otherwise, in agreement with the tumor growth curve, the weight of tumors treated by miR-124 mimic was significantly lower than scramble mimic-injected tumors (Figure 6C). To further identify the role of miR-124 in the suppressive role, we explored the expression of miR-124 and STAT3 in the engrafted tumors. As expected, the expression of miR-124 was significantly up-regulated, while the expression of STAT3 was suppressed in engrafted tumors treated with miR-124 mimic. These data indicated that introduction of miR-124 remarkably inhibited the tumorigenicity of Eca-109 cells in the nude mouse xenograft model, providing a novel method for esophageal cancer therapy.

## Discussion

Although several molecular alterations were identified, esophageal cancers still represent a major challenge of interdisciplinary oncology [24]. Among of which, *STAT3* signaling pathway is particular an important one. *STAT3* always play a critical role in oncogenic signaling in the carcinogenesis and progression of several cancers. In normal cells, STAT3 expression and activity is under tight control to ensure physiological cell proliferation, survival, differentiation and motility. For esophageal cancer, constitutively activated STAT3 expression was found in both esophageal squamous cell carcinomas (ESCC) and Barrett’s adenocarcinomas (BAC) [25]. While, the mechanisms involved in its activation remain to be further identified. Excepts for the potential *STAT3* gene amplification on chromosome 17q21, a region frequently amplified in esophageal adenocarcinomas [26], inflammation-associated STAT3 activation is also conceivable, at least *in vivo* [27]. In this paper, we found another way mediated the overexpression of *STAT3*. The prediction program identified the putative binding site of miR-124 in the 3’UTR of *STAT3*, and we found that overexpression of miR-124 significantly suppressed the expression of *STAT3* and its downstream genes in esophageal cancer cells *in vitro* and engrafted tumors *in vivo*.

Recently, the role of miRNAs in the initiation and maintenance of human diseases has been widely investigated. Considered to be important components of gene regulators, miRNAs play a critical role in the regulation of gene expression and are emerging as novel biomarkers of the diseases [28]. The role of miR-124 has been reported in a variety of cancers, while, so far, few studies addressed miR-124 expression and function in esophageal cancers. Hence, the present study comprehensively addressed these questions in esophageal tissues specimens and cell lines. We found that the majority of esophageal carcinomas showed under-expression of miR-124 (67%). This is consistent with Chen et al., whose work suggested that dysregulation of miR-124 presents borderline longer overall survival and relapse-free survival in acute myeloid leukemia [29]. Otherwise, the expression of miR-124 was reported to be attenuated in human breast cancer tissues, and is reversely correlated with histological grade of the cancer [30]. Herein, we also found some negative relationship between the expression of miR-124 and the clinical and pathological features of esophageal cancer. Although no statistical correlations were observed between miR-124 expression and gender, age, tumor size, TNM stages and metastasis, low level of miR-124 was found to significantly correlate with higher histological grade and tumor location, suggesting that miR-124 might function as a tumor suppressor in esophageal cancer and play a critical role in the progression of esophageal carcinogenesis.

Next, we further explored the comprehensive biological function of miR-124 on the malignant phenotype of esophageal cancer cells. The expression of miR-124 in TE-1 and Eca109 cells were restored using a transient miRNA mimic treatment protocol. As expected, over-expression of miR-124 markedly inhibited cell proliferation, arrested cell cycle progression and induced cell apoptosis of both cell lines. Moreover, miR-124-transfected cells also showed a dramatic decrease in cell migration and invasion. These results shown here demonstrate that miR-124 could suppress the carcinogenesis of esophageal *in vitro*. These results are consistent with Silber et al. reports. They found that miR-124 inhibits cell proliferation *in vitro* and xenograft tumor growth *in vivo* of medulloblastoma cells by targeting cyclin-dependent kinase 6 (CDK6) [31]. In our *in vivo* study, treatment of miR-124 also reduced tumor burden in nude mice, suggesting that miR-124 inhibits the tumor growth of esophageal cancer *in vivo*. Moreover, immunohischemistry assays showed that in the xenografts of mice the expression of STAT3 was significantly suppressed in the miR-124-treated group, which is negatively correlated with the expression of miR-124. These results further identify miR-124 functions as a tumor suppressor in esophageal cancer through, at least partially, targeting *STAT3* signaling pathway. As expected, restoring the expression of *STAT3* in both esophageal cancer cell lines partially abolished miR-124-mediated tumor suppression.

Although we did not explore the role of *STAT3* in esophageal cancer cells in this paper, it has been widely performed by others. Timme et al. reported that STAT3 knockdown reduced cell proliferation and migration of esophageal cancer cells OE33 [25]. A similar study found that STAT3 overexpression affected the proliferation and colony formation of Eca109 cells by altering Erk and Akt activation. *STAT3* regulated the migration and invasion of Eca109 cells independent of Oct-1, while in conjunction with Oct-1, STAT3 inhibited apoptosis of Eca109 cells [10]. Based on the findings that inhibition of *STAT3* resulted in a near complete phenocopy of the effects of miR-124, thus, we speculate that *STAT3* is a central for the suppressive actions of miR-124 in esophageal cancer, which means down-regulation of miR-124 in esophageal cancer cells may contribute to the increased expression of *STAT3* and in turn facilitate the esophageal carcinogenesis.

## Conclusion

Taken together, our results establish a functional link between miR-124 and *STAT3* expression in esophageal cancer, demonstrating that *STAT3* is directly repressed by miR-124, which subsequently inhibits its downstream signaling pathway. Restoring miR-124 function could represent an alternative approach to reduce therapeutically *STAT3* expression, thereby attenuating aggressive tumor properties. Collectively, this finding not only helps us understand the molecular mechanism of esophageal carcinogenesis, but also gives us a strong rationale to further investigate miR-124 as a potential biomarker and therapeutic target for esophageal cancer.

## Abbreviations

STAT: Signal transducer and activator of transcription
miRNA: microRNA
miR-124: microRNA-124
EC: Endometrial carcinoma
ATCC: American type culture collection
DMEM: Dualbecco’s modified eagle’s medium
RT-PCR: Realtime-polymerase chain reaction
FFPE: Formalin-fixed paraffin-embedded
FACS: Fluorescence-activated cell sorting
PI: Propidium iodide
MMP-9: Matrix metallo preteinases-9
ECL: Enhanced chemiluminescence system
ESCC: Esophageal squamous cell carcinomas
BAC: Barrett’s adenocarcinomas
CDK6: Cyclin-dependent kinase 6
